# Role of Glucuronidation for Hepatic Detoxification and Urinary Elimination of Toxic Bile Acids during Biliary Obstruction

**DOI:** 10.1371/journal.pone.0080994

**Published:** 2013-11-14

**Authors:** Martin Perreault, Andrzej Białek, Jocelyn Trottier, Mélanie Verreault, Patrick Caron, Piotr Milkiewicz, Olivier Barbier

**Affiliations:** 1 Laboratory of molecular pharmacology, CHU-Québec Research Centre and the Faculty of Pharmacy, Laval University, Québec, Canada; 2 Department of Gastroenterology, Pomeranian Medical University, Szczecin, Poland; 3 Liver Research Laboratories, Pomeranian Medical University, Szczecin, Poland; 4 Liver Unit, Department of Surgery and Liver Transplantation, Warsaw Medical University, Warsaw, Poland; University of Navarra School of Medicine and Center for Applied Medical Research (CIMA), Spain

## Abstract

Biliary obstruction, a severe cholestatic condition, results in a huge accumulation of toxic bile acids (BA) in the liver. Glucuronidation, a conjugation reaction, is thought to protect the liver by both reducing hepatic BA toxicity and increasing their urinary elimination. The present study evaluates the contribution of each process in the overall BA detoxification by glucuronidation. Glucuronide (G), glycine, taurine conjugates, and unconjugated BAs were quantified in pre- and post-biliary stenting urine samples from 12 patients with biliary obstruction, using liquid chromatography-tandem mass spectrometry (LC-MS/MS). The same LC-MS/MS procedure was used to quantify intra- and extracellular BA-G in Hepatoma HepG2 cells. Bile acid-induced toxicity in HepG2 cells was evaluated using MTS reduction, caspase-3 and flow cytometry assays. When compared to post-treatment samples, pre-stenting urines were enriched in glucuronide-, taurine- and glycine-conjugated BAs. Biliary stenting increased the relative BA-G abundance in the urinary BA pool, and reduced the proportion of taurine- and glycine-conjugates. Lithocholic, deoxycholic and chenodeoxycholic acids were the most cytotoxic and pro-apoptotic/necrotic BAs for HepG2 cells. Other species, such as the cholic, hyocholic and hyodeoxycholic acids were nontoxic. All BA-G assayed were less toxic and displayed lower pro-apoptotic/necrotic effects than their unconjugated precursors, even if they were able to penetrate into HepG2 cells. Under severe cholestatic conditions, urinary excretion favors the elimination of amidated BAs, while glucuronidation allows the conversion of cytotoxic BAs into nontoxic derivatives.

## Introduction

Bile acids (BAs) exert an essential role in the control of lipid, glucose and cholesterol homeostasis (review in [1,2]). Their formation from cholesterol in liver accounts for approximately 90% of total cholesterol catabolism and represents the major driving force contributing to bile formation where the remaining cholesterol is excreted. The primary BAs chenodeoxycholic (CDCA) and cholic (CA) acids, formed from cholesterol in the liver [3], are stored in the gallbladder and secreted in the duodenum [2], where they act as natural detergents to facilitate the absorption of dietary lipids, liposoluble vitamins and cholesterol [2]. The resident bacteria from the large intestine catalyze the 7α-dehydroxylation of CDCA and CA species to generate the secondary BAs, lithocholic (LCA) and deoxycholic (DCA) acids, respectively [3]. Both primary and secondary acids can be reabsorbed and return to the liver *via* the portal circulation. Back in the liver, LCA and CDCA sustain additional biotransformation reactions and are converted into the 6α-hydroxylated hyodeoxycholic (HDCA) and hyocholic acids (HCA), respectively [3,4].

Their detergent properties render BAs cytotoxic at high concentration [5], and their accumulation in liver cells leads to oxidative stress, apoptosis and subsequent damage to the liver parenchyma [3]. Such features are characteristic of cholestatic phenomena, where a reduction of the bile flow limits BA elimination from hepatocytes [6]. Under such conditions, BAs promote hepatocyte necrosis, while favoring apoptosis through the activation of intrinsic and extrinsic pathways [7,8]. BA toxicity is inversely correlated to the number of hydroxyl groups on their sterol backbone [9], and the promotion of less hydrophobic BAs formation has been proposed as a promising therapeutic approach for cholestatic liver diseases [10].

Glucuronidation, catalyzed by UDP-glucuronosyltransferase (UGT) enzymes, is a major detoxification pathway for numerous endo- and xenobiotics [11]. This conjugation reaction transfers a highly hydrophilic glucuronide group to hydrophobic substrates. The resulting glucuronide (G) products are generally more easily excreted and less toxic than the initial molecules (reviewed in 12). Identification of glucuronide conjugate transporters at the basolateral membrane of hepatocytes pointed-out this reaction as a facilitating process for BA secretion into the blood and subsequent urinary elimination [5,12]. Based on this re-routing, BA-conjugating UGT enzymes have been proposed as potential targets to promote BA urinary excretion during cholestasis [12,13]. However, the contribution of glucuronidation to BA detoxification in clinics has received only little attention. Indeed, BA-Gs are rarely investigated in humans, and very little is known about the BA-G urinary levels during cholestasis [14-17]. On the other hand, even if glucuronidation is generally viewed as a detoxification pathway, several examples of glucuronide-induced toxicities have been reported [18]. To the best of our knowledge, the consequences of glucuronidation for BA toxicity have never been analyzed. The present study aimed at evaluating i) the contribution of glucuronidation for urinary BA excretion under a severe cholestatic condition: i.e. biliary obstruction; and ii) whether BA-G species exert cytotoxic effects in the human hepatoma cell line, HepG2.

## Materials and Methods

### Ethics statement

This study was approved by the appropriate clinical study review boards at the CHU de Québec Research Centre, Laval University (“Comité d’éthique de la recherche Clinique du CHUL”, Québec, QC, Canada: projects #95.05.14 and #97.05.14) and the Pomeranian Medical University (Bioethics commission, Pomeranian Medical University in Szczecin, Poland: resolution N° BN-001/43/06). All patients had signed a written consent form before each procedure.

### Materials

Normal and deuterated BAs were purchased from Steraloids Inc. (Newport, RI) and C/D/N Isotopes Inc. (Pointe-Claire, Qc, Canada), respectively. Strata X and Synergie RP Hydro columns were obtained from Phenomenex (Torrance, CA). 3-(4,5-dimethylthiazol-2-yl)-5-(3-carboxymethoxyphenyl)-2-(4-sulfophenyl)-2H-tetrazolium (MTS) reduction 96 Aqueous kit was from Promega (Madison, WI). The Pierce® bicinchoninic assay (BCA) protein assay quantification kit was from Thermo Scientific (Rockford, IL). The Enzchek® caspase-3 assay kit 2 was from Invitrogen (Grand Island, NY). Absorbance and fluorescence were quantified using a Tecan Infinite M1000 series device. Annexin V-FITC and propidium iodine were from eBioscience (San Diego, CA). Protein assay reagents were obtained from Bio-Rad Laboratories Inc. (Marnes-la-Coquette, France). Cell culture reagents were from Invitrogen (Burlington, Canada). HepG2 cells were obtained from the American Type Culture Collection (ATCC, Rockville, MD) and were grown as described [19].

### Patients with biliary obstruction

Urine samples were from 12 patients (6 men and 6 women) with clinical and biochemical features of cholestasis as previously reported [20]. Liver biochemistries, diagnosis, biliary tree dilatation evidences and biliary stenting procedures were extensively described in a previous report [20].

### Bile acid measurement

Bile acid glucuronide concentrations were determined from urine samples (100µL) using liquid chromatography coupled to tandem mass spectrometry (LC-MS/MS) with an electrospray interface, as previously reported [19,21,22]. The chromatographic system consisted of an Alliance 2690 HPLC apparatus (Waters, Milford, MA), and the tandem mass spectrometry system was an API3200 mass spectrometer (Applied Biosystems, Concord, Canada). 

The same urine samples were previously analyzed for unconjugated, taurine-, or glycine-conjugated BAs [20]. In the context of the present study, these concentrations were used to calculate the relative abundance (percentage) of unconjugated, taurine, glycine and glucuronide conjugated BAs.

### Cell viability assays

For MTS reduction assays, hepatoma HepG2 cells were seeded at 20,000 cells/well in 96-well plates, and treated for 48H with increasing BA doses (20 to 200µM). MTS reduction was evaluated using the *CellTiter 96® AQueous One Solution Cell Proliferation Assay* according to the manufacturer’s instructions (Promega).

For total protein determination HepG2 cells were seeded at 5,000cells/well in 96-well plates, and treated for 96H with increasing BA doses (20 to 200µM). Medium was changed each day after a 1X PBS wash in order to remove dead cells. Total protein content was quantified through linear regressions using the Pierce® BCA assay *kit* (Thermo Scientific).

### Quantification of apoptotic and/or necrotic cells

Hepatoma HepG2 cells (400,000 cells/well) were seeded in 6-well plates, and treated with increasing BA concentrations (20 to 200µM) for the indicated duration. Caspase-3 activity was determined using the *EnzChek* Caspase-3 Assay *Kit* (Invitrogen). Apoptotic/necrotic cell quantification was achieved through fluorescence-activated cell sorting (FACS) analyses using the annexin V/propidium iodide-co-labeling method as reported [23]. Labeled cells were then analyzed using a BD FACSCanto II instrument (BD Biosciences).

### Bile acid glucuronides transmembrane transport

Hepatoma HepG2 cells (300,000 cells/well) were seeded in 12-well plates, and exposed to 100µM of LCA-3G, DCA-3G, or CDCA-3G for up to 48H. At each time point, cells were trypsinized and washed with cold solution of 1X PBS/10% FBS, and lysed with 30µL of ultrapure milliQ water. A methanol solution (30µL) containing the deuterated internal standards was then added. Membranes were removed through a 13,000*g* centrifugation after 2 freeze/thaw cycles. Both culture media and cell homogenates were analyzed for BA-Gs using LC-MS/MS.

### Data analysis

Bile acid levels are expressed as mean±SEM. Relative abundance values (expressed as percentages) were calculated as the ratio of unconjugated or conjugated forms divided by the total bile acid concentration. Urinary BAs were not normally distributed according to the Shapiro-Wilk test. The Wilcoxon matched pairs signed-rank test was therefore used for statistical analyses (JMP V7.0.1 program, SAS Institute Inc.).

Statistically significant differences in cell culture experiments were determined through the Student *t* test (JMP V7.0.1 program, SAS Institute Inc.).

## Results

### Biliary stenting reduces urinary levels and improves the relative abundance of bile acid glucuronides in patients with biliary obstruction

In basal cholestatic conditions, urine BA-Gs were more abundant than their parent unconjugated precursors (Table 1). However, under such a severe cholestatic situation, urine samples were also enriched in taurine and glycine conjugates, which were actually 2.4- and 4.0-fold more concentrated than glucuronides, respectively (Table 1). The relative abundances of these BA forms, as calculated for each donor, illustrated the following distribution: glyco-BAs>glucuronide-BAs=taurine-BAs>>unconjugated acids (Table 1). After stenting, the concentration of total BAs (p<0.01), glycine- (p<0.001), taurine (p<0.001) and glucuronide-conjugates were reduced. However, the 74% reduction in BA-G levels failed to reach statistical significance (Table 1). Nevertheless, these changes resulted in major modifications of the composition of the BA pool. Indeed, the relative abundances of tauro- (p<0.05) and glyco-conjugates (p<0.01) were significantly reduced (3- and 4-times, respectively), while those of unconjugated (8-fold) and glucuronides (2-fold) were increased (p<0.05 and p<0.01, respectively).

**Table 1:** Changes of the urinary bile acid pool composition in 12 biliary obstruction patients before and after stenting.

	Before Biliary Stenting	After Biliary Stenting		
Bile acids (µM)	Mean±SEM	Mean±SEM	Fold change	p Value
Unconjugated	0.1±0.1	0.2±0.1	↑ 2	*n.s.*
Glyco-conjugates	18.5±12.2	0.6±0.3	↓ 30	p<0.001
Tauro-conjugates	10.9±8.5	0.3±0.2	↓ 36	p<0.001
Glucuronides	4.8±3.3	1.2±0.1	↓ 4	*n.s.*
**TOTAL**	**34.3±20.3**	**2.3±0.6**	**↓ 15**	**p<0.01**
*Relative abundance (%)^a^*				
Unconjugated	1.6±0.7	12.6±4.4	↑ 8	p<0.05
Glyco-conjugates	45.4±7.5	16.7±4.1	↓ 3	p<0.01
Tauro-conjugates	23.8±4.8	6.6±2.7	↓ 4	p<0.05
Glucuronides	28.9±9.6	63.2±6.8	↑ 2	p<0.01

Unconjugated, taurine, glycine and glucuronide conjugated bile acids were quantified using LC-MS/MS as reported [20] in urine samples (100µL) from 12 stenosed patients (6 ♂ and 6 ♀ , drawn before and after an endoscopic stenting of the bile duct.

The relative abundance (expressed as percentage) of unconjugated, taurine-, glycine- and glucuronide-conjugates were determined by dividing their concentration by the total bile acids.

P-values were determined by the rank sums Wilcoxon matched pairs signed-ranks test: *:p<0.05; ** p<0.01; *** p<0.001. n.s: not significant.

^a^ Data shown represent the mean±SEM of the relative abundance values determined for each sample. The discrepancies between these values and those that can be calculated from the concentrations shown above are caused by the large interindividual variability observed for these compounds.

These results indicate that bile flow interruption causes an accumulation of taurine, glycine and glucuronide conjugates in urine, while after bile flow restoration, this elimination route is mainly used by unconjugated and glucuronidated BAs.

### CDCA, DCA and LCA are cytotoxic for hepatoma HepG2 cells

**Figure 1:**
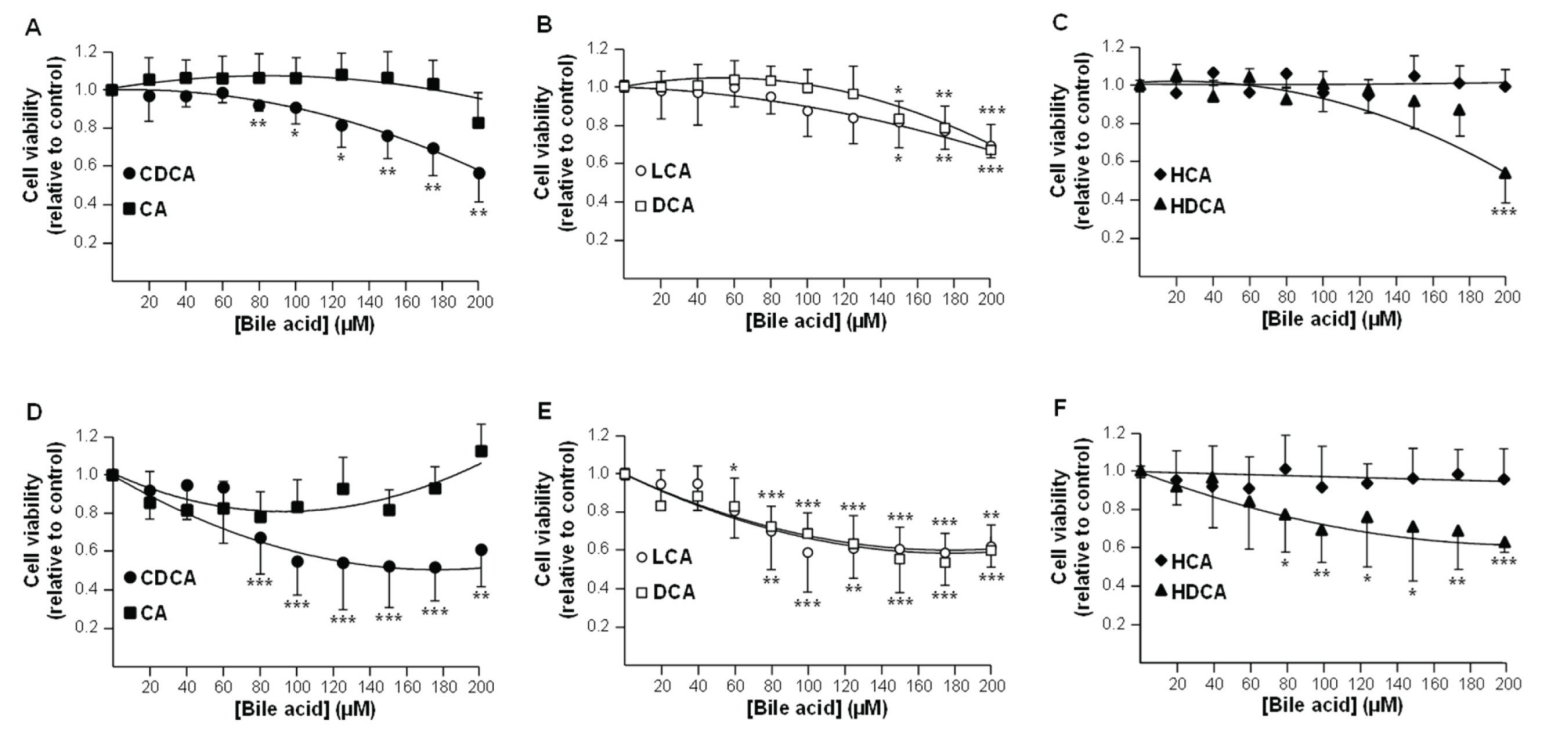
Chenodeoxycholic (A&D), lithocholic (B&E), deoxycholic (B&E) and hyodeoxycholic (C&F) acids reduce HepG2 cell viability. HepG2 cells were exposed to vehicle (DMSO, control) or increasing concentrations of chenodeoxycholic (CDCA, **A & D**), cholic (CA, **A & D**), lithocholic (LCA, **B & E**), deoxycholic (DCA, **B & E**), hyodeoxycholic (HDCA, **C & F**) or hyocholic (HCA, **C & F**) acids for 48 (**A**-**C**) or 96H (**D**-**F**). (**A**-**C**) Cell viability was determined using the MTS reduction assay. (**D**-**F**) The protein content was determined using the Pierce® BCA assay kit. Results, expressed relatively to vehicle-treated (DMSO, control) cells, represent the mean±S.D of 2 independent experiments performed in quadruplicate. Statistically significant differences in vehicle *versus* treated cells were determined using the Student *t* test: *:p<0.05; ** p<0.01; *** p<0.001.

**Figure 2:**
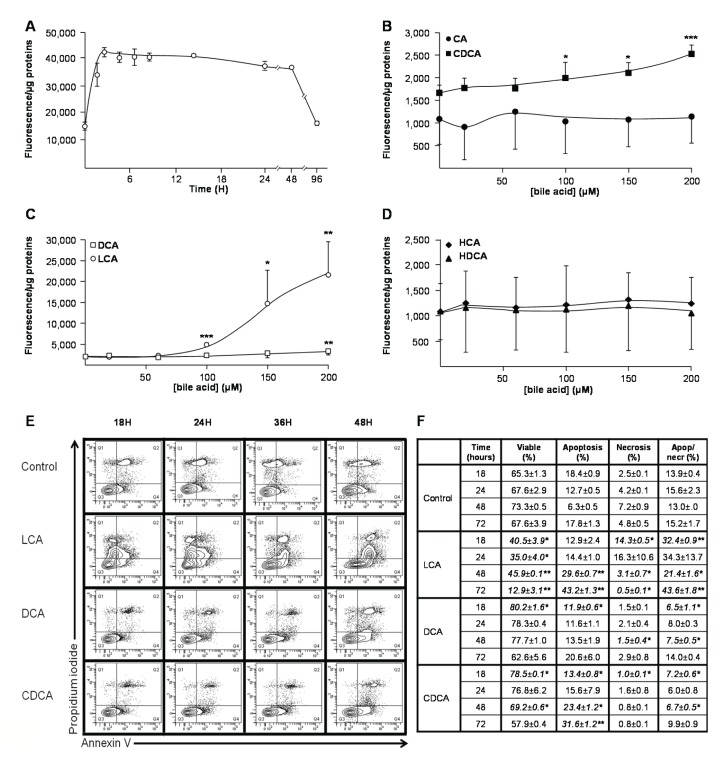
Lithocholic (A,C,E&F), deoxycholic (C,E&F) and chenodeoxycholic (B,E&F) acids promote HepG2 cell death. HepG2 cells were exposed to vehicle (DMSO, control), lithocholic (LCA, **A, C, E & F**), chenodeoxycholic (CDCA, **B, E & F**), cholic (CA, **B**), deoxycholic (DCA, **C, E & F**), hyodeoxycholic (HDCA, **D**) or hyocholic (HCA, **D**) acids. (**A**) HepG2 cells were exposed to DMSO or 100µM LCA for up to 96H, and caspase-3 activity was assessed using the EnzChek Caspase-3 Assay Kit (Invitrogen). (**B**-**D**) HepG2 cells were cultured for 3H in the absence or presence of increasing concentrations (20 to 200µM) of CA (**B**), CDCA (**B**), LCA (**C**), DCA (**C**), HDCA (**D**) or HCA (**D**), and caspase-3 activity was assessed as described in the “Materials and Methods” section. (**E** and **F**) HepG2 cells were exposed to DMSO (Control), 100µM LCA, DCA or CDCA for 18 to 72H. Living, apoptotic and/or necrotic cells were then quantified through fluorescence-activated cell sorting (FACS) analyses using annexin V/propidium iodide-co-labeling, as represented on panel (**E**), and the relative abundance (expressed as percentage) of living (Live), apoptotic (Apop) and/or necrotic (Necr) cell populations were determined by dividing their quartiles by the total cell population (**F**). Statistically significant differences in vehicle *versus* treated cells were determined using the Student *t* test: *:p<0.05; ** p<0.01; *** p<0.001.

**Figure 3:**
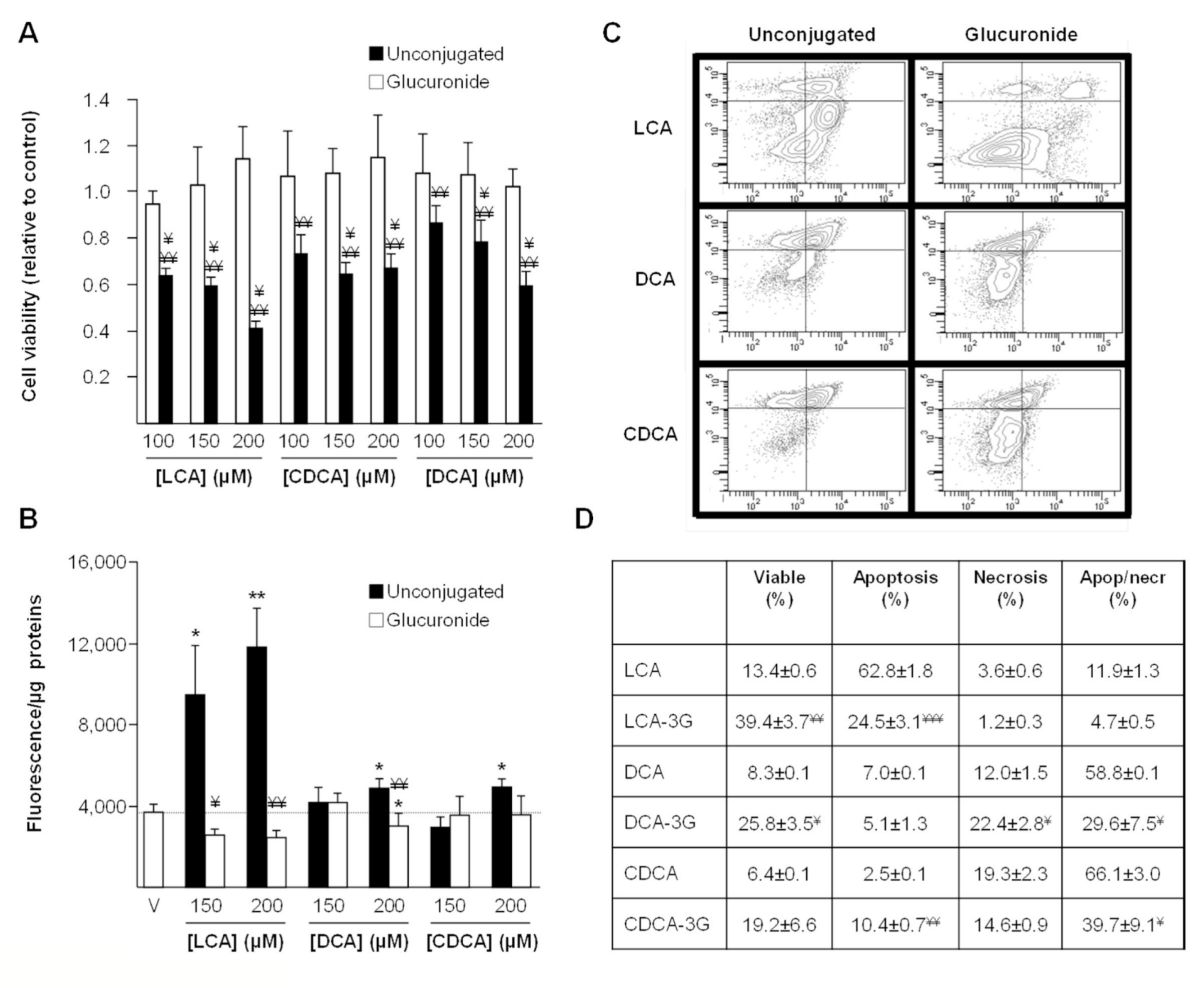
Ether-glucuronide conjugates of lithocholic, deoxycholic and chenodeoxycholic acids are not cytotoxic. (**A** & **B**) HepG2 cells were exposed to vehicle (DMSO, control), or increasing doses (100, 150 and 200µM) of ether-glucuronides or unconjugated forms of lithocholic (LCA), deoxycholic (DCA), or chenodeoxycholic (CDCA) acids for 48 (**A**) or 3H. Cell viability (**A**) was determined using the MTS reduction assay and caspase-3 activity (**B**) was assessed as described in the “Materials and Methods” section. (**C** & **D**) HepG2 cells were exposed to vehicle (DMSO, control), or 200µM LCA, DCA, CDCA, LCA-3G, DCA-3G or CDCA-3G for 24 (LCA/LCA-3G) or 72H (DCA/DCA-3G and CDCA/CDCA-3G). Living, apoptotic and/or necrotic cells were then quantified through fluorescence-activated cell sorting analyses using annexin V/propidium iodide-co-labeling, as represented on panel (**C**), and the relative abundance (expressed as percentage) of living (Live), apoptotic (Apop) and/or necrotic (Necr) cell populations were determined by dividing their quartiles by the total cell population (**D**). Statistically significant differences in vehicle *versus* treated cells (*:p<0.05; ** p<0.01; *** p<0.001) or glucuronide- versus unconjugated-BA treated cells were determined using the Student *t* test: ¥ p<0.05; ^¥¥^:p<0.01; ^¥¥¥^:p<0.001.

**Figure 4:**
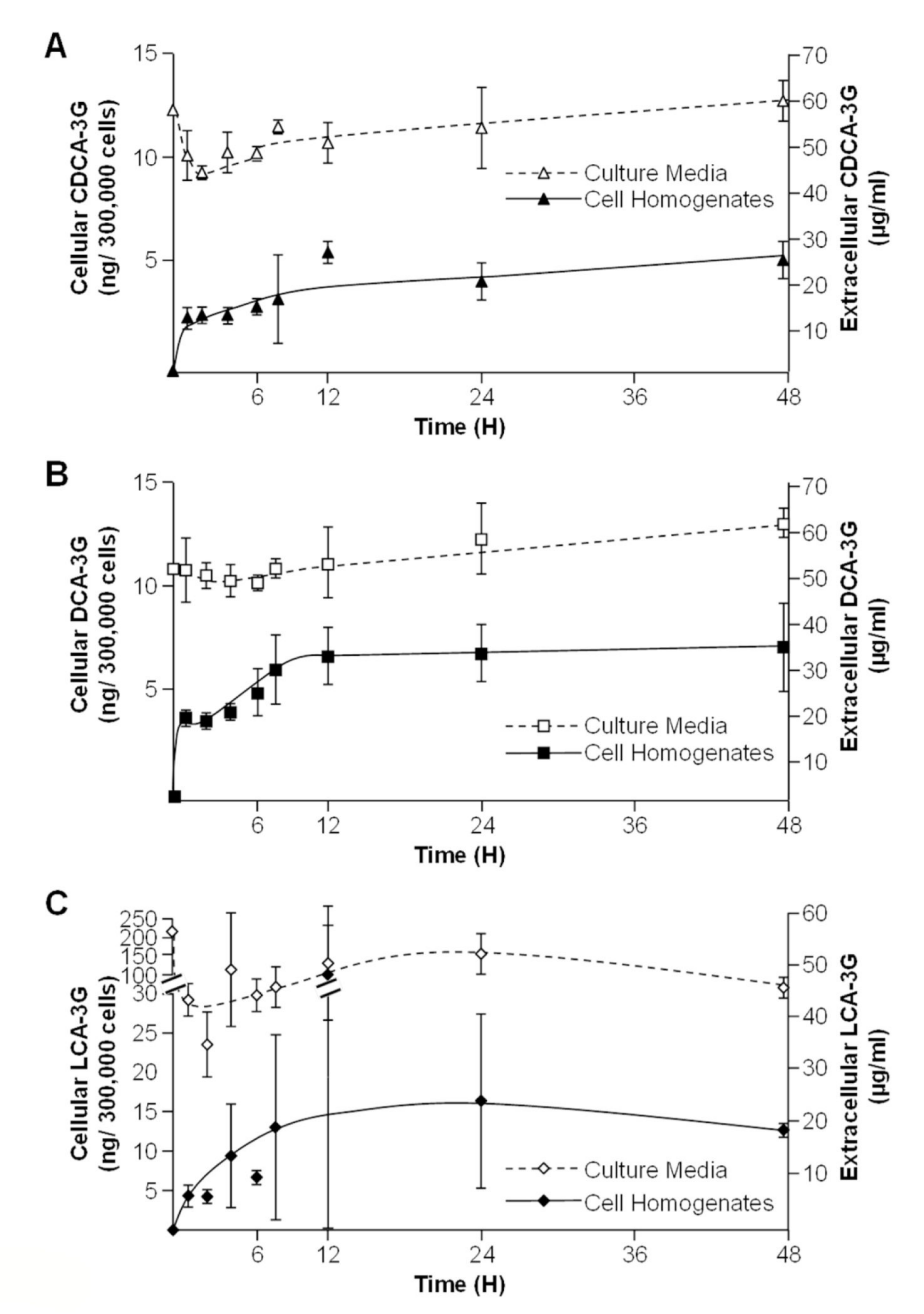
Chenodeoxycholic (A), deoxycholic (B) and lithocholic acid-3glucuronide (C) conjugates are incorporated into HepG2 cells. HepG2 cells were cultured in the presence of vehicle (DMSO, control) or 100µM chenodeoxycholic (CDCA, **A**), deoxycholic (DCA, **B**), or lithocholic (LCA, **C**)- acid 3-glucuronides (G) for 1, 2, 4, 6, 8,12, 24 or 48H. The content in glucuronide conjugates into cell homogenates and culture media was resolved using LC-MS/MS.

We next investigated whether glucuronidation affects BA toxicity in HepG2 cells. For this purpose, we first identified the most cytotoxic unconjugated acids for these cells (Figures 1&2), and then tested the toxic properties of the corresponding ether BA-Gs (Figures 3&4).

In MTS reduction assays, CDCA caused a significant reduction (p<0.01) in HepG2 cells viability after a 48H period of incubation with concentrations as low as 80µM (Figure 1A). LCA, DCA and HDCA also reduced cell viability but only at higher levels (150, 150 and 200µM, respectively) (Figure 1B&C). Similar responses were also observed when cell viability was estimated using total protein content (Figure 1D-F).

A time-course experiment performed with 100µM LCA revealed a significant (p<0.05) and constant increase in caspase-3 activity after only 1H exposure (Figure 2A). Subsequent dose-response analyses, performed for 3H, confirmed LCA as the most pro-apoptotic BA with a significant caspase-3 activation (p<0.001) observed in the presence of more than 100µM (Figure 2C). CDCA and DCA also exhibited pro-caspase effects, but only at higher concentrations (Figure 2B&C). CA, HCA and HDCA were inefficient in modulating this activity (Figure 2B&D). These observations were further confirmed using FACS analyses (Figure 2E&F): LCA caused a significant reduction of viable cells (p<0.05) and an increase of necrotic (p<0.05) and apoptotic+necrotic (p<0.001) cells as soon as after 18H of exposure. While increasing the percentage of viable cells at short term (18H), CDCA also increased the proportion of apoptotic HepG2 cells after 48 and 72H (Figure 2F). Interestingly, in these assays, 100µM DCA did not exhibited convincing cytotoxicity. Actually, this BA caused a reduction in HepG2 cells apoptosis (p<0.05) and apoptosis+necrosis (p<0.05) as well as an increase of the proportion of viable cells (p<0.05) after 18H exposure. Necrotic (p<0.05) and apoptotic/necrotic (p<0.05) cells were also significantly less abundant in DCA-treated cells than in controls, in experiments conducted for 48H. Nevertheless, as for CDCA, exposure to DCA for 48H and 72H also tended to increase the proportion of apoptotic cells. 

Overall, MTS reduction assays and FACS analyses identify CDCA and LCA as the most cytotoxic BA species for hepatoma HepG2 cells. DCA, which reduces HepG2 cells viability in MTS reduction assays, also tended to activate pro-apoptotic events, but only at high concentration.

### Glucuronidated bile acids are not cytotoxic for HepG2 cells

Because BA acyl glucuronides, such as CDCA-, DCA- and LCA-24G are unstable molecules at physiological pH [21], only ether conjugates have been assayed for toxicity.

As illustrated on Figure 3A, the reducing effects of unconjugated acids in MTS reduction assays were loss when these acids were replaced by an equivalent amount of their ether glucuronide derivatives (Figure 3A). In caspase-3 assays, the most impressive changes were observed for LCA-3G (Figure 3B). As indicated above (Figure 2), DCA and CDCA were less pro-apoptotic than LCA, and only stimulated caspase-3 activity at the 200µM dose (Figure 3B). At this concentration, DCA- and CDCA-3G failed to promote cell death (Figure 3B). 

In FACS analyses performed with cells exposed to 200µM unconjugated or glucuronide conjugated BA, CDCA-3G, LCA-3G and DCA-3G left more living cells than their respective unconjugated precursors (Figure 3C&D). However, the most impressive and statistically significant changes were observed for LCA- (p<0.01) and DCA-3G (p<0.05). While the apoptotic cell percentage was reduced in the presence of LCA-3G (p<0.001), apoptosis was found more abundant in cells cultured with CDCA-3G than with CDCA (p<0.01) (Figure 3D). Similar observation also applies to necrosis which was more frequent in cells exposed to DCA-3G than to DCA (p<0.05) (Figure 3D). Beyond these intriguing observations, the proportion of dead cells (apoptosis+necrosis) was significantly reduced when DCA and CDCA were replaced by their respective ether glucuronide conjugates (Figure 3D).

Overall, these observations indicate that glucuronidated bile acids are less toxic than unconjugated BAs for HepG2 cells.

### Glucuronidated bile acids are able to penetrate into HepG2 cells

We next ensured that the low cytotoxic BA-G properties were not only reflecting a reduced cellular capture of these conjugates. HepG2 cells cultured in the presence of 100µM CDCA-, LCA- and DCA-3G for up to 48H, were evaluated for their intra- and extracellular contents in BA-G (Figure 4). All glucuronides were detected in cell homogenates as soon as after a 1H incubation period, and then remained stables for up to 48H (Figure 4). The most impressive accumulation was detected with LCA-3G (Figure 4C). 

These observations therefore indicate that bile acid glucuronides are able to cross the HepG2 cell membrane.

## Discussion

This study establishes the contribution of glucuronidation to bile acid detoxification and urinary elimination when bile flow is interrupted.

Urinary BA-G levels measured in the present study were similar to previous findings [14-16]. The 52.3% of BA-G determined in post-stenting samples resemble to the values reported in urine from healthy women [14], suggesting that bile flow restoration leads to the normalization of urinary BA-G elimination. Accordingly, the 74% reduction observed in post- *vs.* pre-stenting levels is remarkably similar to the 73% difference in urine BA-G levels observed between patients with extrahepatic cholestasis and healthy controls [17]. Therefore, our observations support that urine is an important elimination route for BA-Gs during biliary obstruction. Under normal circumstances, BAs are excreted into the bile at the apical side of hepatocytes [3]. However, by reducing bile flow, biliary obstruction results in a strong accumulation of these natural detergents in liver cells [5,7]. An important consequence of bile acid glucuronidation is the introduction of an additional negative charge to the molecule, which allows their transport by the multidrug resistance related protein (MRP)3 [24]. This transporter, localized in the basolateral membrane of liver cells, facilitates BA-G secretion into the systemic circulation from where they are filtered in the kidney for subsequent elimination in urine [24,25]. Thus, the accumulation of BA-Gs in urine from patients with biliary obstruction reflects the accumulation of unconjugated BAs that occurs in their liver cells.

Nevertheless, when compared to other species, such as taurine and glycine conjugates, the accumulation of BA-G in cholestatic samples appears relatively low. This observation suggests that glucuronide conjugation may not be sufficient by itself to fully detoxify BAs under such severe cholestatic conditions. Previous investigations with the same population revealed a 3-times lower circulating level of unconjugated acids in pre- versus post-stenting serum samples [20]. Considering that these unconjugated acids do not accumulate in the corresponding urine samples, it can be envisioned that their reduced serum concentration actually reflects an improved urinary elimination under the form of glucuronide conjugates. On the other hand, it is also possible that BA-G in urine contributes more efficiently to the elimination of toxic BAs under less severe cholestatic conditions, such as primary biliary cirrhosis (PBC) or primary sclerosing cholangitis (PSC). Actually, these patients present less severe accumulation of blood and urine amidated BAs [26], while their urinary levels of BA-G is also increased [27].

The second aspect of BA-G investigated here relates to their potential toxic properties. While glucuronidation is largely viewed as a detoxification process, several examples of glucuronide-associated cytotoxicities have been reported (reviewed in 18). It was therefore of interest to determine whether BA-Gs are toxic or not for human liver cells. For this purpose, we first established the optimal experimental conditions using unconjugated acids and hepatoma HepG2 cells. Our experiments, which identified CDCA, LCA and DCA as the most toxic BAs, are fully consistent with previous reports in terms of i) differential toxicity of the unconjugated BAs [9,28], ii) cytotoxic doses [9,29,30] and, iii) cell death mechanisms (i.e apoptotic/necrotic effects) [7,31]. More interestingly, the current investigations identify BA-G, such as CDCA-, DCA- and LCA-3G as less cytotoxic forms of bile acids in hepatoma HepG2 cells. This particularly applies to LCA, since its pro-apoptotic effects were drastically reduced when this acids was replaced by its 3glucuronide conjugate. This particularity may actually relate to another unique feature for LCA-3G: i.e its pro-cholestatic effects in rodents [32,33]. Our observations suggest that this cholestatic effect does not reflect an improved hepatic cell death. Actually, we also observe that, within those tested, LCA-3G is the most efficiently up-taken BA-G in HepG2 cells. Thus, one can speculate that when injected to the animals, LCA-3G is efficiently up-taken at the basolateral membrane of hepatocytes and competes with other bile constituents for apical elimination, thus reducing bile formation. Such an hypothesis is supported by the previous finding that LCA-3G has a privileged access to the vesicular transport pathway compared to other bile acid species [34]. On the other hand, DCA- and CDCA-3G exhibited significantly higher pro-necrotic and pro-apoptotic effects than their unconjugated precursors, respectively, in 72H-treated HepG2 cells. This last, and intriguing, observation suggests that the formation of these 2 glucuronide derivatives may be less protective than in the case of LCA-3G. Nevertheless, DCA- and CDCA-3G provoked lower cell death than DCA and CDCA, respectively, confirming their reduced cytotoxicity. Overall, the fact that BA-Gs are non-toxic derivatives supports a protective role for glucuronidation against BA hepatotoxicity. Such a role remains, however, to be confirmed *in vivo* to fully grasp the extent of the protection procured by glucuronidation against toxic bile acids in the whole cholestatic liver. Actually, glucuronidation was previously proposed as being part of a self-protecting mechanism aimed at reducing BA toxicity upon cholestasis (reviewed in 35,36). Such a system involves the BA sensors, farnesoid X-receptor (FXR) and pregnane X-receptor (PXR), which not only control triglycerides, cholesterol and glucose homeostasis, but also modulate the expression of key genes involved in hepatic BA synthesis and metabolism [36]. Indeed, BAs that accumulate in liver cells activate FXR and/or PXR, which in turn down-regulate the expression of the rate-limiting BA-synthesis enzyme cytochrome P450 (CYP)7A1 and up-regulate genes controlling bile acid detoxification: i.ee the BA-conjugating sulfotransferase (SULT)2A1 enzyme, the glucuronosyltransferase UGT2B4, UGT1A3, UGT1A4 enzymes, and the basolateral MRP3, MRP4 and organic solute transporters (OST)α/OSTβ transporters [6,36]. OSTs over-expression facilitates the basolateral removal of amidated acids, while MRPs induction ensures an efficient excretion of sulfate and glucuronide conjugates [35-38]. With such a self-protecting system, glucuronidation was thought to facilitate bile acid removal from hepatic cells [35,37,38]. The present study suggests that improved glucuronidation may also reduce the toxicity of intracellular unconjugated acids during cholestasis.

In conclusion, this study reveals that bile flow interruption causes an accumulation of BA-G in urine. However, due to the huge accumulation of amidated acids occurring under such severe cholestatic conditions, how this improved elimination impacts the extreme BA overcharge remains to be clarified. Nevertheless, the fact that glucuronidated bile acids are non-toxic molecules supports a hepatoprotective role for glucuronidation against cytotoxic unconjugated species such as LCA. Beyond biliary obstruction, future studies will have to determine the contribution of such hepatoprotective properties upon less severe cholestatic conditions, as observed in patients suffering from PBC and PSC [8].
