# Correction: Role of Glucuronidation for Hepatic Detoxification and Urinary Elimination of Toxic Bile Acids during Biliary Obstruction

**DOI:** 10.1371/annotation/ef13ed51-6848-419d-94d8-1bb62e7bcf52

**Published:** 2014-01-06

**Authors:** Martin Perreault, Andrzej Białek, Jocelyn Trottier, Mélanie Verreault, Patrick Caron, Piotr Milkiewicz, Olivier Barbier

A funding organization and grant to the sixth author were incorrectly omitted from the Funding Statement. The Funding Statement should read: "This study was supported by grants from the Canadian Institute of Health Research (CIHR), the Canadian Liver Foundation, the Natural Sciences and Engineering Research Council of Canada and the Canadian Foundation for Innovation, as well as the National Science Centre in Poland (0002011.01/B/NZ5/04216 to P. Milkiewicz).

M. Perreault is holder of a scholarship from the “Fonds pour la Recherche en Santé du Québec” (FRSQ). O. Barbier is holder of a salary grant from CIHR (New investigator #MSH95330). The funders had no role in study design, data collection and analysis, decision to publish, or preparation of the manuscript." 

